# Dietary Supplementation with Conjugated Linoleic Acid Plus *n*-3 Polyunsaturated Fatty Acid Increases Food IntakeBrown Adipose Tissue in Rats

**DOI:** 10.3390/nu1020178

**Published:** 2009-11-26

**Authors:** Alan A. Sneddon, D. Vernon Rayner, Sharon E. Mitchell, Shabina Bashir, Jung-Heun Ha, Klaus W. Wahle, Amanda C. Morris, Lynda M. Williams

**Affiliations:** 1 Vascular Health Programme, Rowett Institute of Nutrition and Health, University of Aberdeen, Aberdeen, AB21 9SB, UK; Email: a.sneddon@abdn.ac.uk (A.A.S.); S.Bashir@abdn.ac.uk (S.B.); 2 Obesity and Metabolic Health Programme, Rowett Institute of Nutrition and Health, University of Aberdeen, Aberdeen, AB21 9SB, UK; Email: rayner@vrayner.plus.com (D.V.R.); s.mitchell@abdn.ac.uk(S.E.M.); honny@kitox.re.kr(J.-H.H.); A.Morris@abdn.ac.uk (A.C.M.); 3 School of Medicine and Dentistry, Cancer Medicine Research Programme, University of Aberdeen, Aberdeen, AB25 2ZD, UK; Email: k.wahle@abdn.ac.uk

**Keywords:** conjugated linoleic acid, *n*-3 long chain polyunsaturated fatty acid, white adipose tissue, brown adipose tissue, hypothalamus, brain

## Abstract

The effect of supplementation with 1% conjugated linoleic acid and 1% *n*-3 long chain polyunsaturated fatty acids (CLA/*n*-3) was assessed in rats. Food intake increased with no difference in body weights. White adipose tissue weights were reduced whereas brown adipose tissue and uncoupling protein-1 expression were increased. Plasma adiponectin, triglyceride and cholesterol levels were reduced while leptin, ghrelin and liver weight and lipid content were unchanged. Hypothalamic gene expression measurements revealed increased expression of orexigenic and decreased expression of anorexigenic signals. Thus, CLA/*n*-3 increases food intake without affecting body weight potentially through increasing BAT size and up-regulating UCP-1 in rats.

## 1. Introduction

There is a great deal of interest in *n*-3 polyunsaturated fatty acids (*n*-3 LC-PUFA) and conjugated linoleic acid (CLA) supplements that may impact on body composition and improve metabolic health. CLA has been reported to have widespread beneficial effects against cancer, atherosclerosis, diabetes and obesity [1] and has been reported to reduce body fat mass in rodents and humans [2,3]. CLA appears to exert its effects on fat mass by redirecting lipid metabolism from white adipose tissue (WAT) to other tissues, predominately the liver, by increasing activity of lipolytic pathways and decreasing lipogenic pathways in WAT [3]. In mice, supplementation with the *trans*-10, *cis*-12-conjugated linoleic acid leads to glucose intolerance, insulin resistance and lipodystrophy and is pro-inflammatory [4]. Conversely, supplementation with the *cis*-9, *trans*-11-CLA increases insulin sensitivity via anti-inflammatory actions [5]. In obese rats a mixture of the two isomers of CLA improves insulin sensitivity, lowers glucose levels and increases adiponectin levels [6], while lean rats do not show these changes [7]. Thus, the effects of CLA in rodents are isomer specific and appear to differ markedly depending on species and adiposity. 

Fish oil derived *n*-3 LC-PUFA have been shown to reduce obesity and to have beneficial effects on glucose and lipid metabolism in rodents [8,9]. Human studies have also shown beneficial effects of *n*-3 LC-PUFA on energy metabolism [10,11] and it has been demonstrated in mice, that *n*-3 LC-PUFA can reverse the deleterious effects of CLA on metabolism, reducing hepatomegaly and liver fat, reversing lipodystrophy and increasing leptin and adiponectin levels [12]. Consequently, it is possible that a combination of CLA plus *n*-3 LC-PUFA could decrease adiposity while avoiding some of the unwanted effects of CLA in other species.

The impact of fatty acids on the hypothalamic centres regulating appetite and energy balance are only now beginning to be investigated [13,14]. The arcuate nucleus (ARC) of the hypothalamus is a key site for the regulation of energy homeostasis [15], and two main regulatory pathways have so far been identified. One pathway consists of neurons co-expressing neuropeptide Y (NPY) and agouti gene-related protein (AgRP), potent stimulators of food intake, while other ARC neurons co-express pro-opiomelanocortin (POMC) and cocaine- and amphetamine-regulated transcript (CART), which suppress food intake [16]. Leptin secreted by, and in proportion to WAT, activates POMC/CART neurons and inhibits NPY/AgRP neurons [17] resulting in inhibition of feeding and an increase in energy expenditure. Leptin acts via the long signalling form of leptin receptor (Ob-Rb) present in the ARC and the ventromedial nucleus (VMH) [18]. However, a number of shorter isoforms of the receptor are present and these are thought to be mainly involved in leptin transport into the brain and are present in the choroid plexus (CP) and brain microvessels [19,20]. Ghrelin, secreted mainly by the stomach acts in opposition to leptin and activates NPY/AgRP neurons [21,22] stimulating feeding and decreasing energy expenditure. Ghrelin acts via the growth hormone secretagogue receptors (GHS-R) present in the ARC and VMH. Long chain fatty acids have been reported to inhibit food intake via a direct effect on hypothalamic energy balance regulatory centres; decreasing NPY and AgRP gene expression levels in the ARC [14,23]. 

We hypothesise that supplementation with a combination of CLA and *n*-3 PUFA may have beneficial additive effects whilst avoiding the unfavorable effect on insulin sensitivity and fatty liver reported for CLA alone. To investigate the outcome of supplementation with CLA plus *n*-3 LC-PUFA on energy balance we fed normal rat chow supplemented with either 1% CLA plus 1% *n-*3 LC-PUFA or 2% vegetable oil to rats for 12 weeks and measured food intake, body weight, WAT, BAT, liver and muscle weights and levels of gene expression in tissues effected by the supplementation, including hypothalamic peptides and receptors known to be involved in energy balance. The ability of leptin to be transported into the brain, potentially regulating leptin signalling to the brain, was also assessed by measuring leptin receptor availability on the CP using [^125^I]leptin binding.

## 2. Results and Discussion

### 2.1. Effects on Food Intake and Body Weight

**Figure 1:**
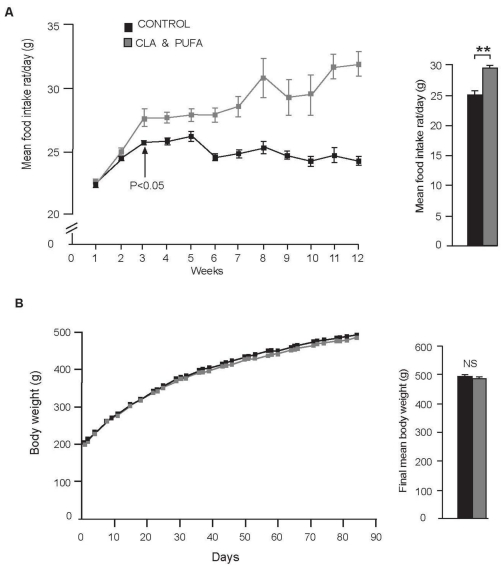
**A.** Mean food intake of rats receiving rat chow supplemented with either 2% vegetable oil (control) or 2% CLA plus *n-*3 LC-PUFA. Mean daily intake per rat was calculated for each week to highlight when significant differences in food intake became apparent *P < 0.05 at three weeks. Daily food intake per rat was also calculated over the 12 weeks of the experiment **P < 0.01 **B.** No significant difference (NS) was found in the body weights of the control supplementation *vs.* the CLA plus *n-*3 LC-PUFA at any of the measurement points during the study or in the final body weights.

Supplementation with CLA plus *n*-3 LC-PUFA compared with control increased food intake, which became significant by the third week of diet (*P* = 0.038). Overall, the average intake of the CLA plus *n-*3 LC-PUFA group was 28 ± 0.5 *vs.* 24.85 ± 0.3 g per rat per day (*P* = 0.004) (Figure 1A). There was no evidence of any difference in body weight between the two groups of rats at any point during the experiment, with the average final weight of CLA plus *n-*3 LC-PUFA animals being 485 ± 6.0 g *vs.* 489 ± 6.6 g for control animals (Figure 1B).

### 2.2. Tissue Weights

**Figure 2:**
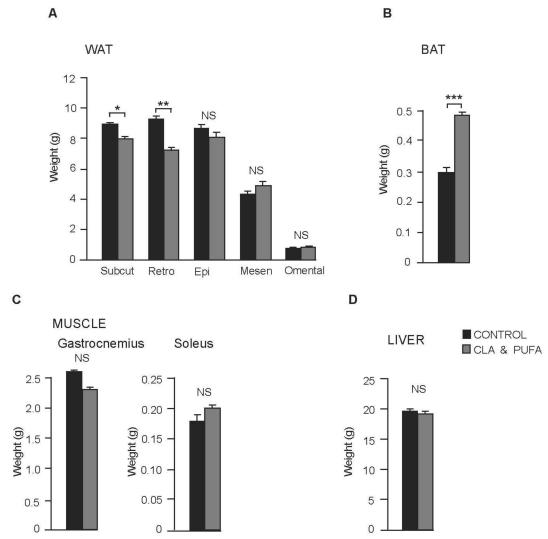
Effect of CLA plus *n-*3 LC-PUFA supplementation on tissue weights. **A.** Retroperitoneal and subcutaneous adipose tissue weights were significantly reduced in the CLA plus *n-*3 LC- PUFA supplemented rats, *P < 0.05, **P < 0.01. There were no significant (NS) changes in the weights of other adipose tissue depots **B.** Subscapular BAT weight was increased, ***P < 0.001. **C.** Weights of gastrocnemius and soleus muscles and **D.** Liver weight was not significantly (NS) changed by CLA plus *n-*3 LC-PUFA supplementation.

Retroperitoneal and subcutaneous adipose tissue weights were significantly reduced in the CLA plus *n-*3 LC- PUFA supplemented rats (–22%, *P* = 0.004, and –13%, *P* = 0.019 respectively, while subscapular BAT was increased by 59% (*P* < 0.001); there were no significant changes in the weights of other adipose tissue depots (Figure 2A and Figure 2B). Weights of liver, gastrocnemius and soleus muscles were not changed by CLA plus *n-*3 LC-PUFA supplementation (Figure 2C and Figure 2D)

### 2.3. Circulating Lipid, Glucose and Hormone Levels

**Figure 3:**
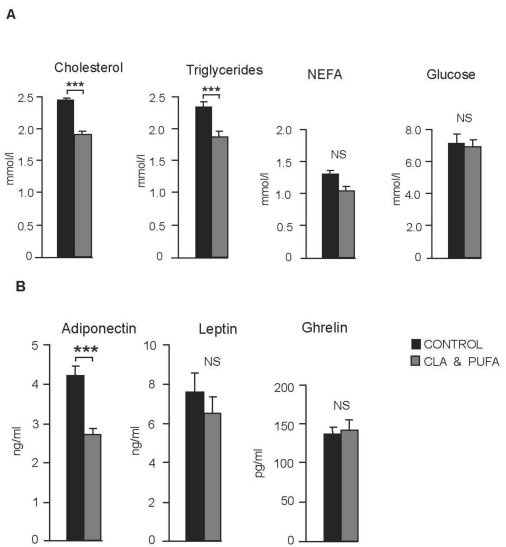
Effect of CLA plus *n-*3 LC-PUFA supplementation on circulating lipid and hormone levels. **A.** CLA plus *n-*3 LC-PUFA supplementation significantly reduced circulating levels of cholesterol and triglyceride, ***P < 0.001 but had no significant (NS) effect on non esterified fatty acid (NEFA) levels or non-fasted glucose levels. **B.** Adiponectin levels were significantly reduced, *P* < 0.001 by CLA plus n-3 LC-PUFA supplementation. There were no significant (NS) changes in plasma ghrelin or plasma leptin levels.

CLA plus *n-*3 LC-PUFA supplementation significantly reduced circulating levels of cholesterol and triglyceride but had no effect on non esterified fatty acid (NEFA) levels or non-fasted glucose levels (Figure 3A). Adiponectin levels were significantly reduced (*P* < 0.001) by CLA plus n-3 LC-PUFA supplementation. There were no significant changes in plasma ghrelin or plasma leptin levels (Figure 3B). 

### 2.4. Liver Lipids and Circulating Liver Enzymes

**Figure 4:**
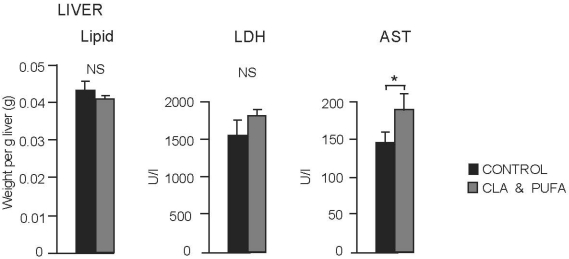
Effect of CLA plus *n-*3 LC-PUFA supplementation on liver lipid content and circulating levels of liver enzymes. Liver lipid levels and lactate dehydrogenase (LDH) were not significantly (NS) changed by supplementation but aspartate aminotransferase activity (AST) was significantly higher in the CLA plus *n-*3 LC-PUFA supplemented animals, *P < 0.05.

There was no effect of CLA plus *n-*3 LC-PUFA supplementation on liver lipid content or circulating levels of lactate dehydrogenase (LDH) but aspartate aminotransferase (AST) activity was significantly higher in the CLA plus *n-*3 LC-PUFA supplemented animals (*P* = 0.049) indicating some degree of liver damage compared to controls (Figure 4 ).

### 2.5. WAT, BAT and Muscle Gene Expression

**Figure 5:**
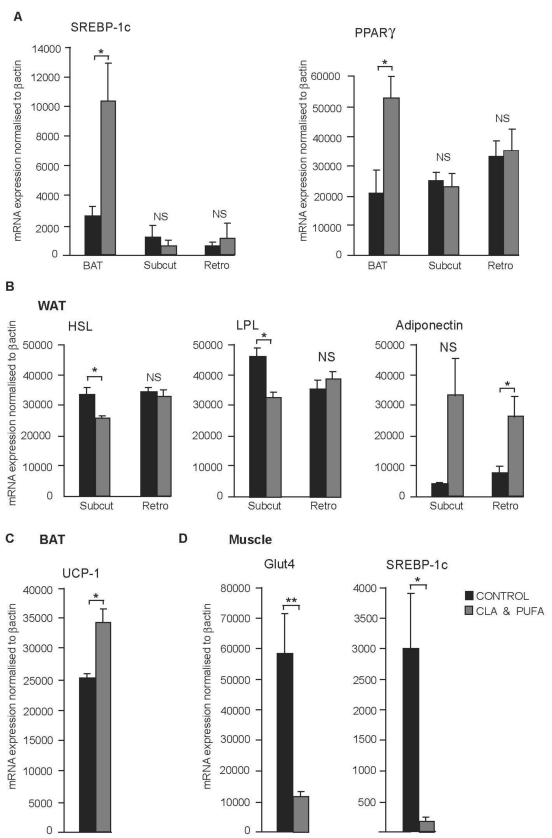
Effect of CLA plus *n-*3 LC-PUFA supplementation on WAT, BAT and muscle gene expression. **A.** There was a significant increase in the expression of SREBP-1c and PPARγ in the BAT of rats receiving the CLA plus *n-*3 LC-PUFA supplementation, *P < 0.05 and no significant (NS) change in either subcutaneous (Subcut) or the retroperitoneal (Retro) WAT. **B**. Hormone sensitive lipase (HSL) and lipoprotein lipase (LPL) were significantly decreased in subcutaneous WAT, *P < 0.05 and were not significantly (NS) changed in retroperitoneal WAT compared with control. Adiponectin gene expression was increased by CLA plus *n-*3 LC-PUFA supplementation in retroperitoneal WAT, * *P* < 0.05 and not significantly (NS) changed in subcutaneous WAT (*P* = 0.09). **C**. UCP-1 expression in BAT was increased by CLA plus *n-*3 LC-PUFA supplementation, *P < 0.05. **D**. There was a significant decrease in the expression of both Glut4 and SREBP-1c in the gastrocnemius muscle of rats receiving the CLA plus *n-*3 LC-PUFA supplementation compared with control animals, **P < 0.01 and *P < 0.05 respectively.

There was a significant increase in the expression of SREBP-1c and PPARγ in the BAT of rats receiving the CLA plus *n-*3 LC-PUFA supplementation (*P* = 0.046 and *P* = 0.032 respectively) (Figure 5A). The expression of these lipid responsive genes did not change in either the subcutaneous or the retroperitoneal WAT depots (Figure 5A). The gene expression of HSL and LPL were significantly decreased in subcutaneous WAT (*P* = 0.022 and *P* = 0.011 respectively) but were unchanged in retroperitoneal WAT. Adiponectin gene expression was increased by CLA plus *n-*3 LC-PUFA supplementation in both the subcutaneous and retroperitoneal depots, but only significantly in the retroperitoneal depot (*P* = 0.09 and *P* = 0.014 respectively) (Figure 5B). The gene expression of UCP-1 in BAT was increased by CLA plus *n-*3 LC-PUFA supplementation (*P* = 0.047) (Figure 5C). Gastrocnemius muscle expression of GLUT4 and SREBP-1c were both down-regulated after CLA plus *n-*3 LC-PUFA supplementation compared to control (*P* = 0.006 and *P* = 0.029, respectively) (Figure 5D).

### 2.6. Hypothalamic Gene Expression and [^125^I]-Leptin Binding

**Figure 6:**
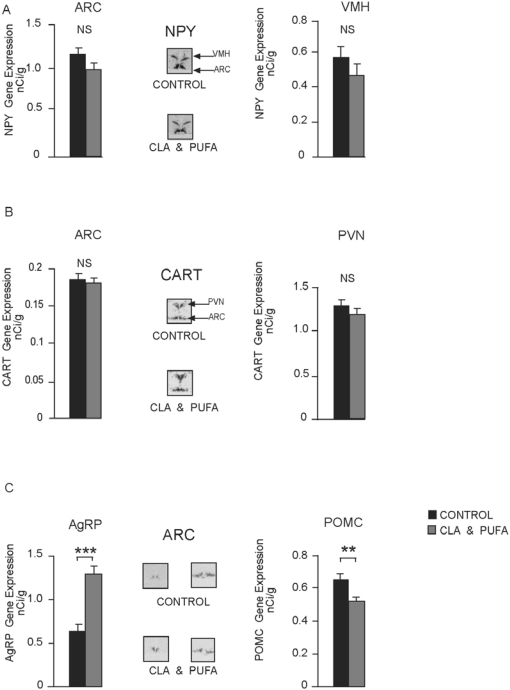
Effect of CLA plus *n-*3 LC-PUFA supplementation on hypothalamic peptide gene expression including representative autoradiographs of the hypothalamic regions measured. **A.** There was no significant (NS) change in NPY or **B.** CART gene expression in either the arcuate nuclei (ARC), ventromedial nuclei (VMH) or paraventricular nuclei (PVN) after supplementation. **C.** AgRP gene expression was increased in the arcuate nuclei (ARC), ***P < 0.001 after CLA plus n-3 LC-PUFA supplementation while POMC gene expression was reduced, **P < 0.005.

**Figure 7:**
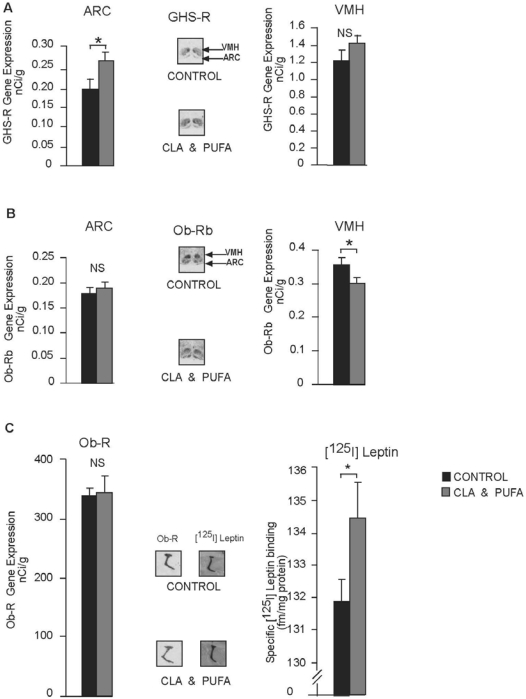
Effect of CLA plus *n-*3 LC-PUFA supplementation on hypothalamic receptor gene expression and [^125^I]leptin binding. **A.** GHS-R was increased in the arcuate nuclei (ARC), *P < 0.05 but not significantly (NS) changed in the ventromedial nuclei (VMH). **B.** Ob-Rb was not significantly (NS) changed in the ARC but significantly decreased in the VMH, *P < 0.05. **C.** There was no significant change (NS) in Ob-R expression in the choroid plexus (CP). The level of specific [^125^I]leptin binding to the CP was increased significantly after CLA plus n-3 LC-PUFA supplementation in both the CP of the lateral ventricles (not shown) and the dorsal third ventricle, *P < 0.05.

There was no significant change in NPY or CART gene expression in either the ARC or the VMH (Figure 6A & Figure 6B). In the ARC, AgRP gene expression was increased (*P* > 0.001) after CLA plus n-3 LC-PUFA supplementation while POMC gene expression was reduced (*P* = 0.007) (Figure 6C). GHS-R was increased in the ARC (*P* = 0.043) but not the VMH, while Ob-Rb was unchanged in the ARC but significantly decreased in the VMH (*P* = 0.049) (Figure 7A & Figure 7B). There were no significant changes in Ob-R expression in the CP. However, the level of specific [^125^I]-leptin binding to the CP was increased significantly after CLA plus n-3 LC-PUFA supplementation in both the CP of the lateral ventricles (LV) (*P* = 0.031) and the dorsal third ventricle (3V) (*P* = 0.019) (Figure 7C).

### 2.7. Discussion

In the present study, CLA plus *n*-3 LC-PUFA supplementation led to a depot specific reduction in WAT with no overall difference detectable in total body weight, an outcome that has been reported previously in studies with CLA and *n*-3 LC-PUFA alone. However, also in the present study, there was a significant increase in food intake in the CLA plus *n*-3 LC-PUFA supplemented animals. This is in contrast to most studies with CLA supplementation alone which have either shown decreased energy intake or no effect [29]. The average daily food intakes did not become significantly different until week three of the experiment, indicating that palatability of the diets was not a major issue. In addition, the loss of adiposity was not accompanied by evidence of a change in hepatic steatosis which has been demonstrated in other rat and mouse CLA studies [30] indicating that lipid storage was not redirected to the liver. Indeed there was no indication of increased liver weight or increased liver triglyceride demonstrated by others [31]; also there was no increase in circulating cholesterol or triglyceride usually associated with increased liver fat storage [31]. There was, however, an increase in circulating levels of AST in the supplemented rats, which may indicate that some liver damage had occurred. 

Food intake is regulated in the hypothalamus by changes in orexigenic and anorexigenic pathways that are modulated by peripheral signals such as leptin, ghrelin and adiponectin as well as by dietary composition [32]. Thus, changes in circulating hormones and the amount and composition of the diet can modulate peptides and receptor levels in hypothalamic nuclei involved in the regulation of intake. In rats, high saturated fat diets have been reported to decrease NPY expression, while high levels of unsaturated fats (both *n*-3 and *n*-6 LC-PUFA) decrease POMC in the ARC [13]. However, in the latter study, the type of fat did not alter hypothalamic gene expression on a low-fat diet. In the present study, the addition of relatively small concentrations of CLA plus *n-*3 LC-PUFA appreciably increased AgRP gene expression in the ARC. AgRP functions by acting as a natural antagonist at the melanocortin 4 receptor (MC4R) blocking the action of the natural agonist α-melanocyte-stimulating hormone, a product of the POMC gene which is down-regulated by CLA plus *n-*3 LC-PUFA supplementation. The up-regulation of the orexigenic signal AgRP and the down-regulation of the anorexigenic POMC gene thus provide a dual signal to increase food intake.

As small amounts of *n-3* LC-PUFA have not been reported to produce any changes in hypothalamic gene expression in the hypothalamic arcuate or ventromedial nuclei in rats [13], the changes seen with CLA plus *n-*3 LC-PUFA must result either from the CLA alone or a synergistic action between CLA and *n-*3 LC-PUFA. Whether these changes are the result of a direct effect of the CLA plus *n-*3 LC-PUFA on hypothalamic neurons or are the result of the diminished WAT signalling back to the hypothalamus is unclear. However, levels of circulating leptin appear to be minimally lowered or unchanged after CLA plus *n-*3 LC-PUFA supplementation, ruling out WAT-derived leptin as a possible mechanism. However, there may be other circulating factors from WAT, not measured in the present study, which may signal the brain. Similarly, there is no difference in the level of circulating ghrelin. However, [^125^I]-leptin binding to the choroid plexus was significantly increased in the supplemented rats indicating that the transport of leptin into the brain may be increased. Nonetheless, the difference was relatively small and the biological impact of such a change is unknown. Leptin acts as a powerful anorexigenic agent in lean animals. Increased leptin transport into the brain should not result in the gene changes observed in the hypothalamus or in the increased food intake exhibited. In addition, circulating adiponectin, which acts centrally to increase food intake, is reduced by approximately 40% with CLA plus *n-*3 LC-PUFA supplementation. Therefore, the drop in circulating adiponectin also cannot give rise to the changes in gene expression seen in the hypothalamus or the increased food intake. 

Decreased circulating levels of adiponectin have previously been shown in mice fed for 22 days with levels of CLA and fish oil similar to those used in the present study [12]. However, the fact that circulatory adiponectin levels were decreased at the same time as adiponectin gene expression was increased in the two WAT depots tested in the present study indicates that the mixture of CLA plus *n-*3 LC-PUFA has a complex effect. In a previous study *n-*3 LC-PUFA was shown to increase adiponectin gene expression and adiponection protein content of WAT in *ob/ob* mice [33]. In contrast, CLA has been shown to inhibit the secretion of adiponectin from cultured rat adipocytes [34]. These findings taken together provide an explanation for the apparent discrepancy in the present study where a combination of CLA plus *n-*3 LC-PUFA was used. 

The increase in food intake without an associated increase in body weight may only be explained by an increase in energy expenditure in the CLA plus *n-*3 LC-PUFA treated animals. Physical activity measurements were not carried out in the present study, but analysis of gene expression in gastrocnemius muscle showed decreased levels of both GLUT4 and SREBP-1c mRNA (by 5-fold and 10-fold, respectively) in the rats supplemented with CLA/n-3 PUFA. These results suggest that rats in this group did not display increased muscle locomotor activity, as increased levels of activity have been shown to be associated with increased expression of GLUT4 [35] and SREBP-1c [36]. This also means that the increased size of the BAT depot together with increased BAT UCP-1 expression remains as the sole potential mechanism identified in the present study whereby at least some of the energy is dissipated. BAT has high uptake rates for both glucose and lipids and regulates energy expenditure through the activation of UCP-1 which generates heat via lipid oxidation. An increase in BAT thermogenesis could explain the ability of the animals to eat more while maintaining their body weight. However, the effect of supplementation on the body temperature of the rats was not measured in the present study. Whereas previous studies with CLA supplementation alone have shown conflicting effects of CLA on BAT size and UCP-1 expression [37,38,39], *n*-3 PUFA have been shown to both increase BAT UCP-1 levels [40] and to induce BAT thermogenic activity [41]. Furthermore, as CLA alone has been shown to suppress the expression and activity of PPARγ, [42], a transcription factor which plays a central role in the differentiation of both white and brown adipose cells [43,44], it seems likely that increased PPARγ expression drives BAT enlargement in the CLA plus *n-*3 LC-PUFA supplemented animals. 

CLA plus *n*-3 supplementation also invoked a depot specific reduction in both retroperitoneal and subcutaneous WAT. Such a heterogeneous reduction in adiposity has been observed in many different animal models and is a classical characteristic of CLA supplementation alone where it appears to be the result of decreased lipid accumulation within adipocytes [29]. One of the key enzymes in lipid metabolism is adipocyte LPL and the inhibition of LPL activity has been significantly correlated with the suppressing effect of CLA on lipogenesis [45]. Therefore, at least in the case of subcutaneous WAT, the reduction in adiposity induced by CLA plus *n*-3 supplementation in this depot was correlated with decreased LPL expression. 

Taken together these data show that CLA plus *n-*3 LC-PUFA supplementation decreases WAT size without increasing liver lipid content. However, unexpectedly food intake was increased in the supplemented rats, which was reflected by an increase in hypothalamic orexigenic and a decrease in anorexigenic gene expression. This may be explained by the increase in the size of the BAT depot and an increase in UCP-1 activity in this tissue. As both CLA and *n-*3 LC-PUFA are readily available as supplements in humans and BAT has been identified in adult humans [46] the potential impact of supplementation with CLA plus *n-*3 LC-PUFA on human BAT remains an interesting possibility that requires further study.

## 3. Experimental Section

### 3.1. Experimental Animals and Supplementation

All studies involving animals were licensed under the Animal (Scientific Procedures) Act of 1986 and received approval from the Rowett Research Institute’s Ethical Review Committee. Male Hooded Lister rats (Rowett strain) aged 10 weeks were housed in groups of four per cage. Cages were then randomly allocated to one of two groups (n = 12 in each group) and fed isocalorific diets containing standard rodent laboratory chow (SDS, Essex, UK) supplemented with either 1% CLA (Clarinol G-80 containing 760 mg CLA/g consisting of a 50:50 mixture of *cis*-9, *trans*-11: *trans*-10, *cis-*12 CLA from Loders Croklaan, Wormerveer, the Netherlands) plus 1% *n*-3 LC-PUFA (EPAX 5500TG; 280 mg EPA and 180 mg DHA/g from Pronova Biocare a.s. Lysaker, Norway) or 2% vegetable oil (Rathburn Chemicals, Walkerburn, Scotland). The oils were added to pellets and allowed to absorb. The pellets were then purged with nitrogen and frozen at –20 °C, to prevent oil oxidation, until required. Animals were fed *ad libitum* and fresh food was provided every other day and uneaten food discarded. Daily total food intake for each cage was recorded. Food spillage was also measured. There was no evidence of differential spillage between the diets. Animals were weighed three times per week. After 12 weeks, rats were killed by stunning and cervical dislocation prior to decapitation. Trunk blood was collected into heparin coated tubes and centrifuged at 1,000 g and stored at –80 °C until required. The WAT depots; epididymal, retroperitoneal, subcutaneous, mesenteric and omental as well as BAT, liver and the gastrocnemius and soleus muscles were dissected, weighed and frozen in liquid nitrogen and stored at –80 °C. The whole brain was removed and frozen on dry ice and stored at –80 °C. 

### 3.2. Plasma and Liver Lipids, Glucose and Circulating Liver Enzymes

All measurements including circulating levels of aspartate aminotransferase (AST) and lactate dehydrogenase (LDH) were carried out by Analytical Services at the Rowett Institute of Nutrition and Health, using a Thermo Konelab 30 clinical analyser and the appropriate kits supplied by Thermo Kone (Manchester, UK). Liver lipids were extracted and measured using a chloroform/methanol extraction according to the method of Bligh and Dyer [24]. 

### 3.3. Semi Quantitative RT-PCR

Total RNA was isolated using tissue depots, from three representative animals from each treatment, using Trizol (Invitrogen, Paisley, UK) according to the manufacturers’ instructions. RNA integrity and concentration were measured using the Agilent bioanalyser (Agilent Technologies UK Ltd, Stockport, UK). cDNA was prepared using the Protoscript cDNA synthesis kit (ABgene, Epsom, UK) using 1μg total RNA and a 2720 thermocycler (Applied Biosystems, Warrington, UK). Genes were amplified using the following primers: β-actin (forward primer: 5’-AGC ACA ATG AAG ATC AAG AT, reverse primer: 5’-TGT AAC GCA ACT AAG TCA TA); uncoupling protein-1 (UCP-1) (forward primer: 5’-ATA CTG GCA GAT GAC GTC CC, reverse primer: 5’-ATC CGA GTC GCA GAA AAG AA); lipoprotein lipase (LPL) (forward primer: 5’- CCT GAA GAC TCG CTC TCA GA, reverse primer: 5’-TTG GTT TGT CCA GTG TCA GC); hormone sensitive lipase (HSL) (forward primer: 5’-AGG ACA CCT TGG CTT GAG CG, reverse primer: 5’-TGC CCA GGA GTG TGT CTG AG); peroxisome proliferator-activated receptor-γ (PPARγ) (forward primer: 5’-TGA TAT CGA CCA GCT GAA CC, reverse primer: 5’-GTC CTC CAG CTG GTT CGC CA); sterol regulatory element binding protein-1c (SREBP-1c) (forward primer: 5’-GCG GAG CCA TGG ATT GCA C, reverse primer: 5’-CTC TTC CTT GAT ACC AGG CCC); Adiponectin (forward primer: 5’-GGA ACT CGA GTG TCA CGA TG, reverse primer: 5’-TGG CAG CCT TCA GGA ACC CT); GLUT4 (forward primer: 5’-TCT CGG TGC TCT TAG TAG, reverse primer: 5’-CCA ATC TCA AAG AAG GCC ACA AA). All genes were amplified by heating at 94 °C, for 4 min, then 94 °C for 45 sec, 60 °C for 45 sec, 72 °C for 45 sec, for 25 cycles (β-actin), for 30 cycles (UCP-1, LPL, HSL), for 40 cycles (Adiponectin, GLUT4) or for 50 cycles (PPARγ), except SREBP-1c which was amplified by heating at 94 °C for 4 min, then 94 °C for 45 sec, 52 °C for 45 sec, 72 °C for 45 sec, for 30 cycles. PCR products of the predicted size were quantified on agarose gels using the UviPhoto gel documentation system (Uvitec Ltd, Cambridge, UK) and AIDA software (Raytest, Straubenhardt, Germany). For each amplified sequence, the number of PCR cycles was previously determined to lie within the linear range (data not shown). For normalization, β-actin levels were used as an internal control.

### 3.4. In Situ Hybridization

Specific probes for GHS-R, Ob-R, Ob-Rb, AgRp, NPY, CART and POMC were used as detailed previously [25,26,27]. Automated sequencing was performed to verify each sequence. Messenger RNA levels were quantified using [^35^S] labelled riboprobes and *in situ* hybridisation, on 20µm thick coronal hypothalamic sections, using techniques described in detail elsewhere [28]. Briefly, slides were fixed in 4% (w/v) paraformaldehyde in phosphate buffered saline (PBS) (0.1 mol/L phosphate buffer/0.9% (w/v) saline) for 20 min at room temperature, washed in PBS, incubated in 0.1 mmol/l triethanolamine for 2 min and acetylated in 0.1 mmol/L triethanolamine and 0.25% (v/v) acetic anhydride for 10 min. Sections were dehydrated in ethanol and dried under vacuum before hybridisation with [^35^S]-labelled riboprobes at 10^6^ cpm/ml for 18 h at 58 °C. After hybridisation, sections were desalted through a series of washes in standard saline citrate (SSC) to a final stringency of 0.1 X SSC at 60 °C for 30 min, treated with RNase A and dehydrated in ethanol. Slides were apposed to Biomax MR (Sigma, Poole, Dorset, UK) together with [^14^C] micro-scale standards (Amersham International, Amersham, UK) at room temperature for varying lengths of time depending on the probes used. 

### 3.5. In vitro Autoradiography

Sections were acid prewashed in low pH, high salt (pH 2, 0.5M NaCl) HEPES buffer, to remove any endogenous leptin bound to its receptor, prior to a brief wash in HEPES before incubation with 1nM [^125^I]leptin with the specific activity adjusted to 250,000 cpm/pM in HEPES for 2 h at room temperature. Control slides were incubated with [^125^I]leptin as above plus 1μM leptin. Slides were then thoroughly washed in HEPES buffer followed by distilled water at 4°C and air dried before apposed to Biomax MR (Sigma, Poole, Dorset, UK) together with [^125^I] micro-scale standards (Amersham International, Amersham, UK) at room temperature.

### 3.6. Quantification of in situ Hybridisation and in vitro Autoradiography

Autoradiographs were scanned on a Umax Power Look II (UMAX Data Systems, Fremont, CA, USA). Integrated optical densities (IOD), a measurement of both surface area and optical density of images, were quantified using the Image Pro-plus system (Media Cybernetics, Silver Springs, MD, USA) and converted to nCi/g using [^14^C] microscale standard curves. For quantification of [^125^I]leptin binding to the choroid plexus, average optical densities were measured. Average optical densities were converted to nCi/g using [^125^I] microscale standard curves.

### 3.7. Plasma Hormones

Serum leptin and ghrelin concentrations were measured using commercially available rat-specific radioimmunoassay kits (Linco Research Inc., St. Charles, MI, USA and Phoenix Diagnostics Inc., Belmont, CA, USA, respectively). Adiponectin was measured by ELISA (B-Bridge International, CA, USA).

### 3.8. Statistical Analysis

Data are represented as mean ± SEM and were analysed by one-way ANOVA. *P* = 0.05 was considered statistically significant. 

## 4. Conclusions

This study shows that the CLA plus *n-*3 LC-PUFA supplementation is effective in decreasing WAT deposition without an increase in liver lipid. Unexpectedly this decrease in WAT was accompanied by an increase in food intake. The increase in food intake appears to be due to an increase in hypothalamic orexigenic and a decrease in anorexigenic gene expression. The increased food intake does not result in an increase in body weight due to activation of BAT. This is particularly important when the occurrence and function of BAT in adult humans has been identified [46]. Further studies are required to identify how the combination of CLA plus *n-*3 LC-PUFA drives these changes in the hypothalamus and BAT.

## References

[1] Wahle K.W., Heys S.D., Rotondo D. (2004). Conjugated linoleic acids: are they beneficial or detrimental to health?. Prog. Lipid. Res..

[2] Blankson H., Stakkestad J.A., Fagertun H., Thom E., Wadstein J., Gudmundsen O. (2000). Conjugated linoleic acid reduces body fat mass in overweight and obese humans. J. Nutr..

[3] House R.L., Cassady J.P., Eisen E.J., McIntosh M.K., Odle J. (2005). Conjugated linoleic acid evokes de-lipidation through the regulation of genes controlling lipid metabolism in adipose and liver tissue. Obesity Rev..

[4] Poirier H., Shapiro J.S., Kim R.J., Lazar M.A. (2006). Nutiritional supplementation with trans-10, cis-12-conjugated linoleic acid induces inflammation of white adipose tissue. Diabetes.

[5] Moloney F., Toomey S., Noone E., Nugent A., Allan B., Loscher C.E., Roche H.M. (2007). Antidiabetic effects of cis-9, trans-11-conjugated linoleic acid may be mediated via anti-inflammatory effects in white adipose tissue. Diabetes.

[6] Houseknecht K.L., Vanden Heuvel J.P., Moya-Camarena S.Y., Portocarrero C.P., Peck L.W., Nickel K.P., Belury M.A. (1998). Dietary conjugated linoleic acid normalizes impaired glucose tolerance in the Zucker diabetic fatty fa/fa rat. Biochem. Biophys. Res. Commun..

[7] Noto A., Zahradka P., Ryz N.R., Yurkova N., Xie X., Taylor C.G. (2007). Dietary conjugated linoleic acid preserves pancreatic function and reduces inflammatory markers in obese, insulin-resistant rats. Metabolism.

[8] Ikemoto S., Takahashi M., Tsunoda N., Maruyama K., Itakura H., Ezaki O.  (1996). High-fat diet-induced hyperglycemia and obesity in mice: Differential effects of dietary oils. Metabolism-Clin. Exp..

[9] Ruxton C.H.S., Reed S.C., Simpson M.J.A., Millington K.J. (2004). The health benefits of omega-3 polyunsaturated fatty acids: a review of the evidence. J. Hum. Nutr. Diet..

[10] Delarue J., Li C.H., Cohen R., Corporeau C., Simon B. (2006). Interaction of fish oil and a glucocorticoid on metabolic responses to an oral glucose load in healthy human subjects. Brit. J. Nutr..

[11] Lombardo Y.B., Chicco A.G. (2006). Effects of dietary polyunsaturated n-3 fatty acids on dyslipidemia and insulin resistance in rodents and humans. A review. J. Nutr. Biochem..

[12] Ide T. (2005). Interaction of fish oil and conjugated linoleic acid in affecting hepatic activity of lipogenic enzymes and gene expression in liver and adipose tissue. Diabetes.

[13] Dziedzic B., Szemraj J., Bartkowiak J., Walczewska A. (2007). Various dietary fats differentially change the gene expression of neuropeptides involved in body weight regulation in rats. J. Neuroendocrinol..

[14] Obici S., Feng Z.H., Morgan Y., Stein D., Karkanias G., Rossetti L. (2002). Central administration of oleic acid inhibits glucose production and food intake. Diabetes.

[15] Kalra S.P., Dube M.G., Pu S., Xu B., Horvath T.L., Kalra P.S. (1999). Interacting appetite-regulating pathways in the hypothalamic regulation of body weight. Endocr. Rev..

[16] Williams G., Bing C., Cai X.J., Harrold J.A., King P.J., Liu X.H. (2001). The hypothalamus and the control of energy homeostasis Different circuits, different purposes. Physiol. Behav..

[17] Elias C.F., Aschkenasi C., Lee C., Kelly J., Ahima R.S., Bjorbaek C., Flier J.S., Saper C.B., Elmquist J.K. (1999). Leptin differentially regulates NPY and POMC neurons projecting to the lateral hypothalamic area. Neuron.

[18] Mercer J.G., Hoggard N., Williams L.M., Lawrence C.B., Hannah L.T., Trayhurn P. (1996). Localization of leptin receptor mRNA and the long form splice variant (Ob-Rb) in mouse hypothalamus and adjacent brain regions by *in situ* hybridization. FEBS Lett..

[19] Bjorbaek C., Elmquist J.K., Michl P., Ahima R.S., van Bueren A., McCall A.L., Flier J.S. (1998). Expression of leptin receptor isoforms in rat brain microvessels. Endocrinology.

[20] Bjorbaek C., Uotani S., da Silva B., Flier J.S. (1997). Divergent signaling capacities of the long and short isoforms of the leptin receptor. J. Biol. Chem..

[21] Kamegai J., Tamura H., Shimizu T., Ishii S., Sugihara H., Wakabayashi I. (2000). Chronic central infusion of ghrelin increases hypothalamic neuropeptide Y and agouti-related protein mRNA levels and body weight in rats. Diabetes.

[22] Cowley M.A., Smith R.G., Diano S., Tschop M., Pronchuk N., Grove K.L., Strasburger C.J., Bidlingmaier M., Esterman M., Heiman M.L., Garcia-Segura L.M., Nillni E.A., Mendez P., Low M.J., Sotonyi P., Friedman J.M., Liu H.Y., Pinto S., Colmers W.F., Cone R.D., Horvath T.L. (2003). The distribution and mechanism of action of ghrelin in the CNS demonstrates a novel hypothalamic circuit regulating energy homeostasis. Neuron.

[23] Morgan K., Obici S., Rossetti L. (2004). Hypothalamic responses to long-chain fatty acids are nutritionally regulated. J. Biol. Chem..

[24] Bligh E.G., Dyer W.J. (1959). A rapid method of total lipid extraction and purification. Can. J. Biochem. Physiol..

[25] Nogueiras R., Tovar S., Mitchell S.E., Rayner D.V., Archer Z.A., Dieguez C., Williams L.M. (2004). Regulation of growth hormone secretagogue receptor gene expression in the arcuate nuclei of the rat by leptin and ghrelin. Diabetes.

[26] Adam C.L., Moar K.M., Logie T.J., Ross A.W., Barrett P., Morgan P.J., Mercer J.G. (2000). Photoperiod regulates growth, puberty and hypothalamic neuropeptide and receptor gene expression in female Siberian hamsters. Endocrinology.

[27] Mercer J.G., Moar K.M., Ross A.W., Hoggard N., Morgan P.J. (2000). Photoperiod regulates arcuate nucleus POMC, AGRP, and leptin receptor mRNA in Siberian hamster hypothalamus. Am. J. Physiol..

[28] Mitchell S.E., Robinson J.J., King M.E., McKelvey W.A.C., Williams L.M. (2002). Interleukin 8 in the cervix of non-pregnant ewes. Reproduction.

[29] Wang Y.W., Jones P.J. (2004). Conjugated linoleic acid and obesity control: efficacy and mechanisms. Int. J. Obes. Relat. Metab. Disord.

[30] Purushotham A., Shrode G.E., Wendel A.A., Liu L.F., Belury M.A. (2007). Conjugated linoleic acid does not reduce body fat but decreases hepatic steatosis in adult Wistar rats. J. Nutr. Biochem..

[31] Koba K., Akahoshi A., Yamasaki M., Tanaka K., Yamada K., Iwata T., Kamegai T., Tsutsumi K., Sugano M. (2002). Dietary conjugated linolenic acid in relation to CLA differently modifies body fat mass and serum and liver lipid levels in rats. Lipids.

[32] Lopez M., Tovar S., Vazquez M.J., Williams L.M., Dieguez C. (2007). Peripheral tissue-brain interactions in the regulation of food intake. Proc. Nutr. Soc..

[33] Gonzalez-Perez A., Horrillo R., Ferre N., Gronert K., Dong B., Moran-Salvador E., Titos E., Martinez-Clement M., Lopez-Parra V.A., Claria J. (2009). Obesity-induced insulin resistance and hepatic steatosis are alleviated by ω-3 fatty acids: a role for resolvins and protectins. FASEB J..

[34] Perez-Matute P., Marti A., Martinez J.A., Fernandez-Otero M.P., Stanhope K.L., Havel P.J., Moreno-Aliaga M.J. (2007). Conjugated linoleic acid inhibits glucose metabolism, leptin and adiponectin secretion in primary cultured rat adipocytes. Mol. Cell Endocrinol..

[35] Holloszy J.O., Hansen P.A., Blaustein M.P., Grunicke H., Habermann E., Pette D., Schultz G., Schweiger M. (1996). Reviews of Physiology, Biochemistry and Pharmacology: Regulation of glucose transport into skeletal muscle.

[36] Ikeda S., Miyazaki H., Nakatani T., Kai Y., Kamei Y., Miura S., Tsuboyama-Kasaoka N., Ezaki O. (2002). Up-regulation of SREBP-1c and lipogenic genes in skeletal muscles after exercise training. Biochem. Biophys. Res. Commun..

[37] Ryder J.W., Portocarrero C.P., Song X.M., Cui L., Yu M., Combatsiaris T., Galuska D., Bauman D.E., Barbano D.M., Charron M.J., Zierath J.R., Houseknecht K.L. (2001). Isomer-specific antidiabetic properties of conjugated linoleic acid. Improved glucose tolerance, skeletal muscle insulin action, and UCP-2 gene expression. Diabetes.

[38] West D.B., Blohm F.Y., Truett A.A., DeLany J.P. (2000). Conjugated linoleic acid persistently increases total energy expenditure in AKR/J mice without increasing uncoupling protein gene expression. J. Nutr..

[39] Sugano M., Akahoshi A., Koba K., Tanaka K., Okumura T., Matsuyama H., Goto Y., Miyazaki T., Murao K., Yamasaki M., Nonaka M., Yamada K. (2001). Dietary manipulations of body fat-reducing potential of conjugated linoleic acid in rats. Biosci. Biotechnol. Biochem..

[40] Takahashi Y., Ide T. (2000). Dietary n-3 fatty acids affect mRNA level of brown adipose tissue uncoupling protein 1, and white adipose tissue leptin and glucose transporter 4 in the rat. Brit. J. Nutr..

[41] Oudart H., Groscolas R., Calgari C., Nibbelink M., Leray C., Le Maho Y., Malan A. (1997). Brown fat thermogenesis in rats fed high-fat diets enriched with n-3 polyunsaturated fatty acids. Int. J. Obes. Relat. Metab. Disord.

[42] Takahashi Y., Kushiro M., Shinohara K., Ide T. (2002). Dietary conjugated linoleic acid reduces body fat mass and affects gene expression of proteins regulating energy metabolism in mice. Comp. Biochem. Physiol. B Biochem. Mol. Biol..

[43] Barak Y., Nelson M.C., Ong E.S., Jones Y.Z., Ruiz-Lozano P., Chien K.R., Koder A., Evans R.M. (1999). PPAR gamma is required for placental, cardiac, and adipose tissue development. Mol. Cell.

[44] Nedergaard J., Petrovic N., Lindgren E.M., Jacobsson A., Cannon B. (2005). PPARgamma in the control of brown adipocyte differentiation. Biochim. Biophys. Acta.

[45] Park Y., Storkson J.M., Albright K.J., Liu W., Pariza M.W. (1999). Evidence that the trans-10,cis-12 isomer of conjugated linoleic acid induces body composition changes in mice. Lipids.

[46] Nedergaard J., Bengtsson T., Cannon B. (2007). Unexpected evidence for active brown adipose tissue in adult humans. Am. J. Physiol..

